# Does Nursing Home Residents’ Right to Self-Determination Improve Their Quality of Life in South Korea?

**DOI:** 10.1093/geroni/igaa057.294

**Published:** 2020-12-16

**Authors:** Minhong Lee, Kyeongmo Kim, Sok An

**Affiliations:** 1 Dong-eui University, Busan, Pusan-jikhalsi, Republic of Korea; 2 Virginia Commonwealth University, Richmond, Virginia, United States; 3 Korea Rural Economic Institute, Austin, Texas, United States

## Abstract

Background and Purpose: Addressing issues of quality of life of nursing home residents based on the human rights-based approach has been a top priority in the long-term care system in Korea but no study has yet examined the relationship between self-determination of nursing home residents and their quality of life. This study aimed to examine whether greater levels of self-determination in the provision of daily care were associated with higher levels of quality of life of the residents. Methods: We collected data from 332 residents (+65) at 20 nursing homes in a metropolitan city. We measured residents’ right to self-determination using the autonomy scale of the Client-centered Care Questionnaire. We also included quality of life, socio-economic characteristics, ADLs, depressive symptoms, and social networks. We ran multiple regression analysis using SPSS 26.0. Results: The findings of this study revealed that greater levels of residents’ right to self-determination were associated with higher levels of quality of life (β =-.425, p <.0001). Older residents who were higher levels of depressive symptoms were likely to have lower levels of quality of life (β = -.265, p < .001). Conclusions and Implications: This study adds to the growing literature on the ways nursing home residents’ self-determination contributes to their quality of life. More opportunities for self-determination in their treatment should be given to promote recovery and to encourage participation in the decision-making process. Nursing practitioners and policymakers in Korea should develop programs and/or services that enhance residents’ self-determination to improve their quality of life.

